# Novel Valve-in-Valve Transcatheter Systemic Tricuspid Valve Replacement in Congenitally Corrected Transposition of the Great Arteries

**DOI:** 10.1016/j.jaccas.2024.102736

**Published:** 2024-12-04

**Authors:** Aamer Naofal, Riyad Yazan Kherallah, Wilson W. Lam, Dhaval R. Parekh

**Affiliations:** a Department of Medicine, Baylor College of Medicine, Houston, Texas, USA; b Division of Cardiology, Department of Medicine, Baylor College of Medicine, Houston, Texas, USA; c Division of Cardiology, Department of Pediatrics, Baylor College of Medicine, Houston, Texas, USA

**Keywords:** CCTGA, congenitally corrected transposition of the great arteries, tricuspid stenosis, valve-in-valve

## Abstract

A 41-year-old woman with history of situs inversus totalis, dextrocardia, congenitally corrected transposition of the great arteries, and systemic tricuspid valve replacement presented with worsening of her baseline orthopnea and exertional dyspnea caused by stenosis of the tricuspid valve. Given her high risk for surgery, valve-in-valve transcatheter tricuspid valve replacement was pursued and performed successfully, leading to resolution of her symptoms. Previous valve-in-valve tricuspid interventions are rare. This case is notable as the first systemic atrioventricular valve-in-valve replacement in a patient with dextrocardia through a septal approach.

## Visual Summary

**Figure:**
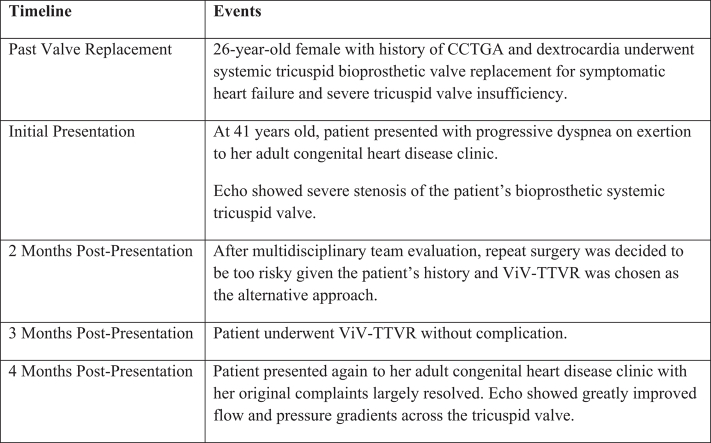

**Visual Summary:**
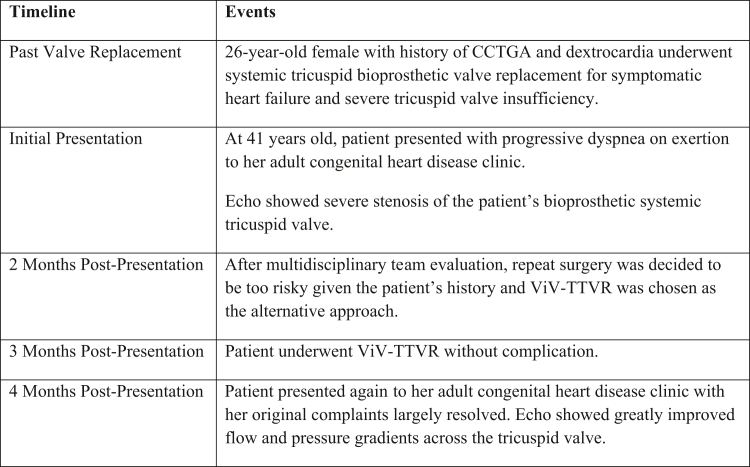
Case Timeline Events summarizing this case. CCTGA = congenitally corrected transposition of the great arteries; Echo = echocardiography; ViV-TTVR = valve-in-valve transcatheter tricuspid valve replacement.

## History of Presentation

A 41-year-old woman with a history of congenitally corrected transposition of the great arteries (CCTGA) and systemic tricuspid bioprosthetic valve replacement presented with reports of progressive dyspnea on exertion, orthopnea, and bendopnea. She was unable to perform activities she once enjoyed such as dancing and noted significant shortness of breath, such as having to stop after walking up one-half a flight of stairs.

**Take-Home Messages**

• CCTGA subjects the morphologic right ventricle to systemic pressures, potentially leading to tricuspid valve disease that may necessitate multiple interventions over time.
• ViV-TTVR is an innovative procedure that can be used in patients who experience complications of their systemic tricuspid valve replacement and who are deemed to be at high risk for surgery.

## Past Medical History

**Figure 1:**
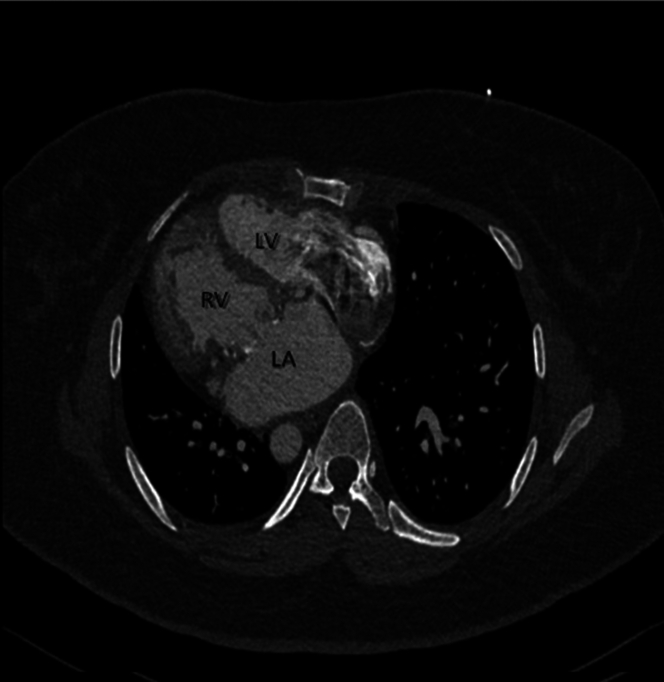
Computed Tomography Scan The scan demonstrates the unique cardiac anatomy of the patient at the level of the tricuspid valve. LA = left atrium; LV = left ventricle; RV = right ventricle.

**Figure 2:**
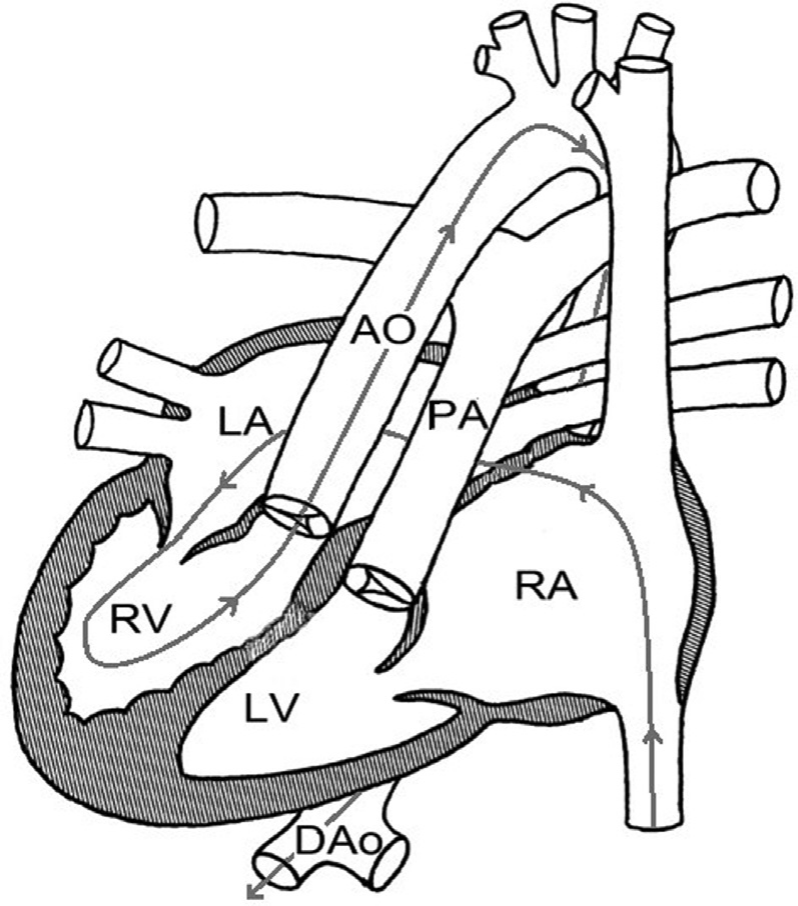
Mullins Diagram The cardiac anatomy of a patient with congenitally corrected transposition of the great arteries and dextrocardia. This anatomy is marked by mirror atrial arrangement, discordant atrioventricular connections, right-handed ventricular topology, and an anterior and rightward oriented aorta (Ao). Additionally, the pathway of the rail used for valve placement is clearly marked with arrows. DAo = descending aorta; PA = pulmonary artery; RA = right atrium; other abbreviations as in Figure 1.

The patient had a past history of situs inversus totalis with dextrocardia and CCTGA with a history of systemic tricuspid valve replacement with a 33-mm Biocor bioprosthetic valve (St Jude Medical) for severe tricuspid valve insufficiency at 26 years of age (Figures 1 and 2).

## Differential Diagnosis

Important considerations for the differential diagnosis included sequelae of CCTGA. Right ventricular dysfunction can result from prolonged exposure to systemic pressures, compounded by potential perfusion mismatches from the single coronary artery supplying the systemic right ventricle. CCTGA is often accompanied by comorbid anomalies, including ventricular septal defects, pulmonary stenosis, and variations in the systemic atrioventricular valve. Complete heart block is prevalent given the atypical positioning of the atrioventricular node and His bundle. Pulmonary hypertension is a common cause of dyspnea in patients with uncorrected congenital heart disease. Finally, the patient’s history of tricuspid valve replacement over 15 years ago raises concern for structural valve degeneration of the tricuspid valve.

## Investigations

**Figure 3:**
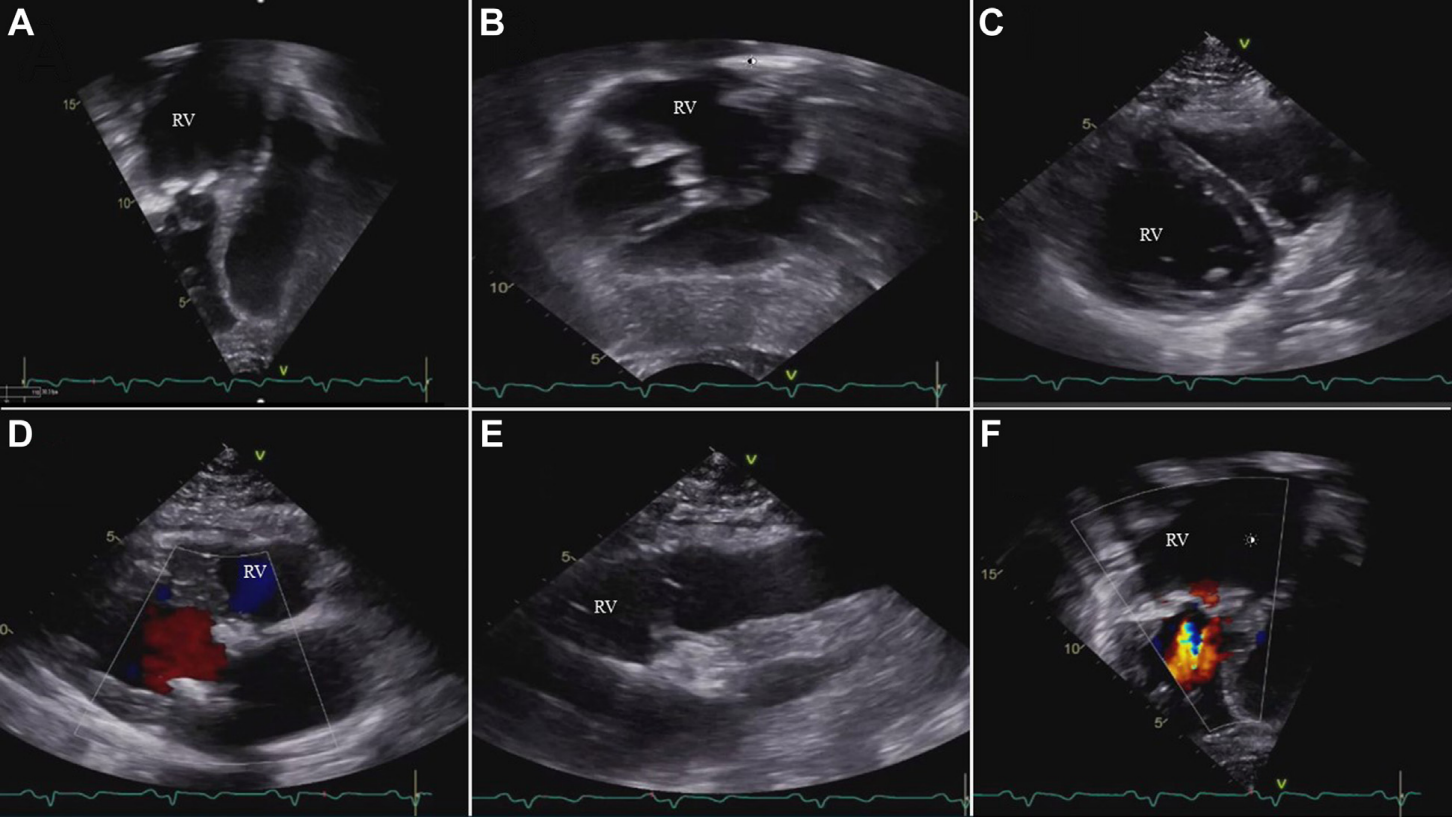
Transthoracic Echocardiography: Multiple Views The following views demonstrate systemic right ventricular hypertrophy, mild to moderate systolic dysfunction, and a stenotic appearing tricuspid valve. (A) 4-chamber view. (B) Subcostal view. (C) Short-axis view. (D) Long-axis view. (E) Long-axis aorta view. (F) Turbulent flow in the 4-chamber view. RV = right ventricle.

Transthoracic echocardiography (TTE) revealed significant stenosis of the patient’s bioprosthetic systemic tricuspid valve, evidenced by a mean gradient of 18 mm Hg at a heart rate of 68 beats/min. Notably, there was an absence of tricuspid regurgitation. Additionally, the assessment indicated qualitative mild to moderate systolic dysfunction of the right ventricle, whereas left ventricular systolic function remained normal (Figures 3A to 3F).

## Management

**Figure 4:**
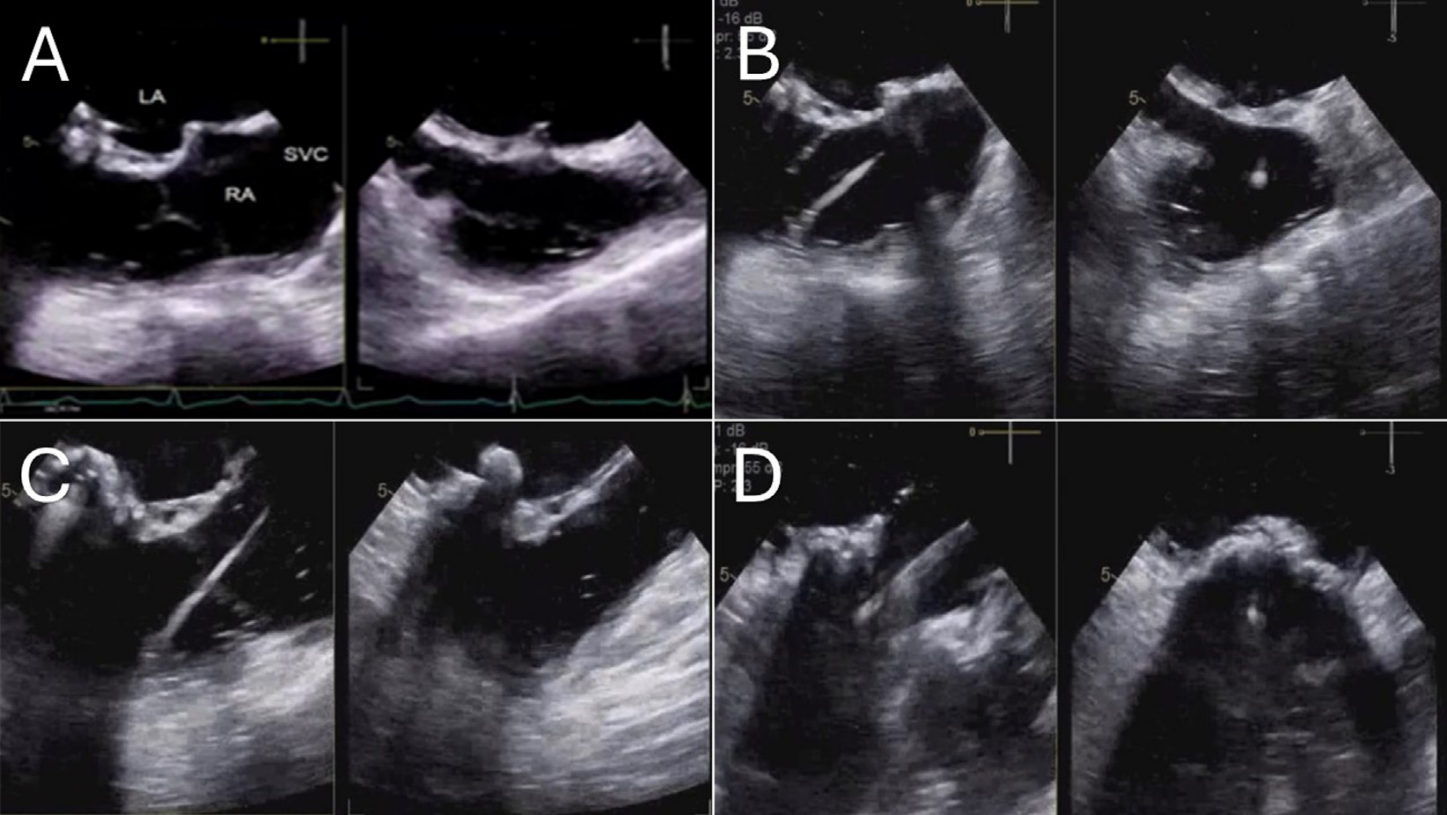
Transesophageal Echocardiography: Multiple Views These views, captured at each step of the procedure, outline the process of placing the new valve. (A) Transseptal puncture. (B) Advancement of the pacing wire. (C) Rail placement for valve deployment. (D) Positioning and ballooning of the stented valve. SVC = superior vena cava; other abbreviations as in Figures 1 and 2.

**Figure 5:**
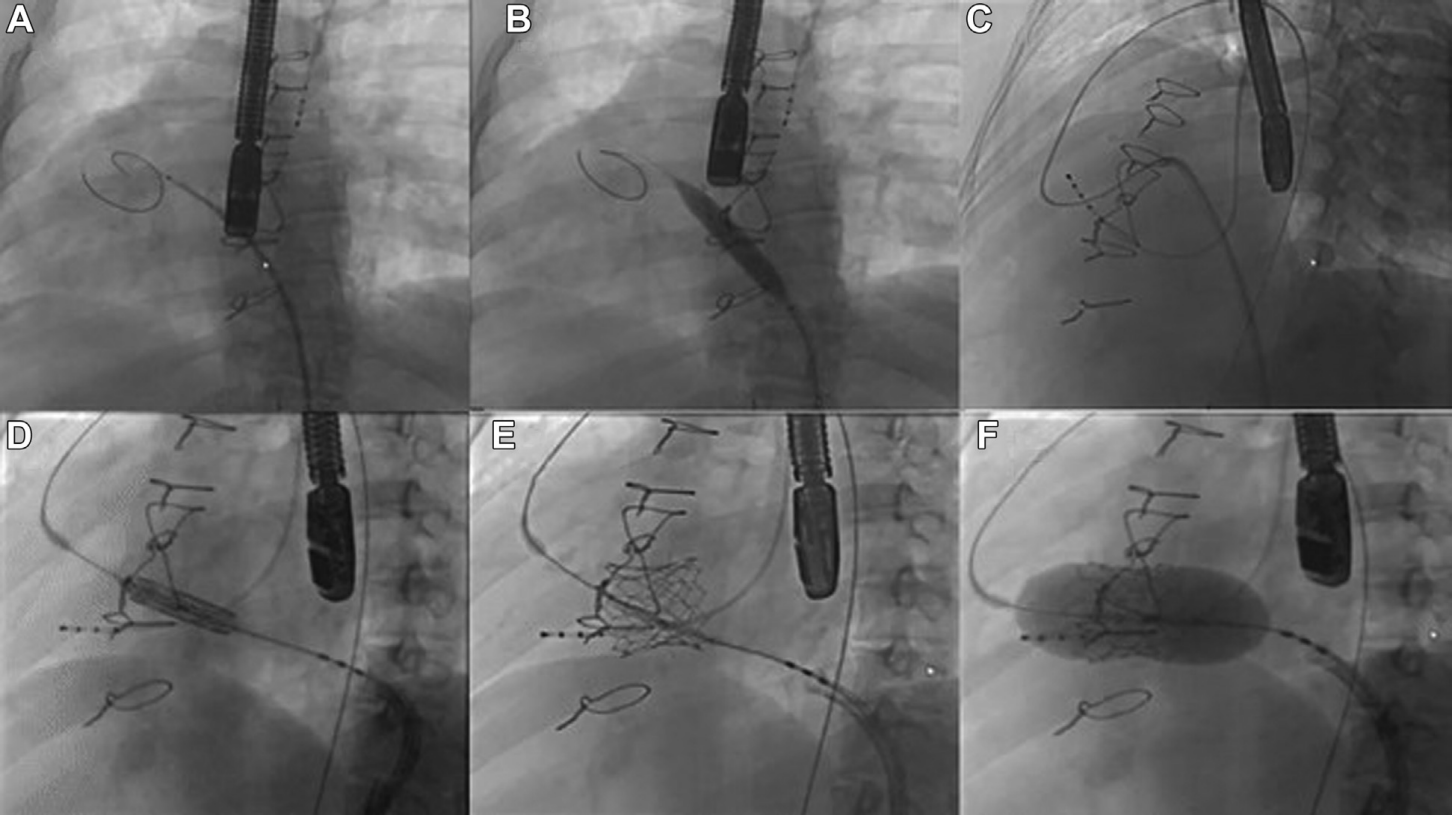
Fluoroscopic View of Deployment of Valve-in-Valve Transcatheter Tricuspid Valve Replacement Key stages of the procedure. (A and B) Achievement of transseptal puncture. (C) Advancement of the Amplatzer Super Stiff wire (Boston Scientific) to establish an adequate rail. (D) Positioning of the valve before deployment. (E and F) Final deployment of the 29-mm Edwards Sapien 3 valve (Edwards Lifesciences).

**Figure 6:**
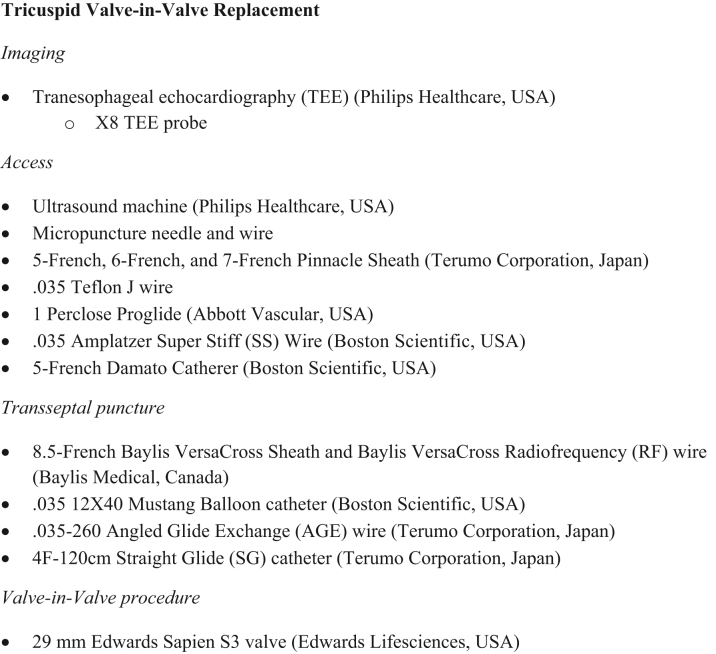
Equipment List This list contains the equipment used to perform the valve-in-valve transcatheter tricuspid valve replacement.

Multidisciplinary team evaluation deemed her to be at high surgical risk because of her age, previous sternotomies, and right ventricular dysfunction. The decision was made to pursue valve-in-valve transcatheter tricuspid valve replacement (ViV-TTVR), which was performed under transesophageal echocardiography (TEE) and fluoroscopic guidance, with modifications to standard guiding views on the basis of the patient’s anatomy. Access was obtained with a 24-F DrySeal sheath (W.L. Gore & Associates) in the left femoral vein and a 6-French sheath in the right femoral artery. The fossa of the interatrial septum was engaged with a rightward and anterior approach with the 8.5-F Baylis VersaCross sheath (Baylis Medical) and the Baylis VersaCross radiofrequency wire (Baylis Medical) (Figures 4A to 4D). Care was taken to position the puncture high enough to allow the undeployed valve to sit fully in the left atrium, thereby preventing interaction with the atrial septum during subsequent positioning and deployment. To accommodate the 29-mm Edwards Sapien 3 valve and its delivery sheath (Edwards Lifesciences), predilation with a 12 mm × 40 mm Mustang Balloon catheter (Boston Scientific) was then performed at nominal pressure (Figures 5A and 5B). At this stage, to ensure for secure anchoring and stability of the delivery system, the Baylis VersaCross was exchanged over the 4-F 120-cm Straight Glide catheter (Terumo Corporation) for a 0.035-inch 260-cm Angled Glidewire Exchange wire (Terumo Corporation). This wire was maneuvered through the aortic valve into the descending aorta, and a 0.035-inch Amplatzer Super Stiff (Boston Scientific) wire was positioned distally with ample purchase to deliver the valve appropriately and was snared with a 25-mm goose neck snare from the right femoral artery sheath to provide an adequate rail. It was necessary to use such a rail because the distance from the tricuspid valve to the right ventricular apex did not allow for enough wire purchase to support the Edwards Valve and delivery system (Figure 5C). Subsequently, a 29-mm Edwards Sapien 3 valve was advanced through the left femoral access, delivered transseptally, and placed across the systemic tricuspid valve (Figures 5D to 5F, Videos 1 and 2). The formal list of equipment required to perform the ViV-TTVR is listed below (Figure 6). Pressure measurements in the right-sided left atrium and systemic right ventricle revealed no residual pressure gradient across the systemic tricuspid valve. TEE confirmed adequate positioning without perivalvular leak (Video 3).

## Outcome and Follow-Up

At 1-year follow-up, the patient’s symptoms completely resolved. One-year TTE showed a mean gradient of 5 mm Hg across the ViV-TTVR, moderate right ventricular hypertrophy with qualitatively low-normal systolic function, and trivial regurgitation.

## Discussion

Clinically significant tricuspid regurgitation is prevalent in patients with CCTGA, affecting approximately 20% to 50% of individuals.^1^ Right ventricular dysfunction arises when the morphologic right ventricle is subjected to systemic pressures. This can lead to progressive functional tricuspid valve regurgitation secondary to annular dilation, leaflet tethering, and geometric distortion. Additionally, the tricuspid valve may have Ebsteinoid dysplasia marked by failed leaflet delamination from the right ventricular wall and apical displacement. Tricuspid regurgitation may be both a contributor to and a consequence of right ventricular dysfunction, creating a vicious cycle.

Two main approaches are used in addressing CCTGA.^2^ Whereas anatomical repair attempts to restore the normal anatomical relationship between the left and right ventricles with the systemic and pulmonary circulation, physiologic repair maintains the right ventricle as the systemic ventricle and corrects physiologic abnormalities as they manifest. Anatomical repair is the preferred option in early life for individuals with a compatible morphologic left ventricle. Physiologic repair, however, remains an important option for patients when the left ventricle is not suitable or preconditioned to support the systemic circulation.

Because patients with complex congenital heart disease are living longer, the lifelong management of valvular heart disease is becoming increasingly relevant. In patients with bioprosthetic valvular degeneration, transcatheter treatment plays an important role by offering a minimally invasive option for patients at high operative risk and minimizing the lifetime number of redo sternotomies. Experience in adult congenital heart disease (ACHD) is relatively limited, but various different approaches have been undertaken. Previous reports highlighted successful tricuspid edge-to-edge repair for atrioventricular regurgitation in a range of conditions, including Fontan circulation and CCTGA.^3^^,^^4^ In the valve-in-valve international data registry, in which approximately one-half of the cohort had a history of congenital heart disease, ViV-TTVR was associated with excellent rates of technical success and great clinical and hemodynamic outcomes at 3-year follow-up.^5^ Additionally, a single-center series study demonstrated favorable evidence of positive cardiac remodeling post–ViV-TTVR in patients with ACHD at up to 5 years.^6^

Our patient’s dextrocardia introduced an additional layer of complexity that demanded an experienced imaging and interventional team. Previous successful interventions in patients with dextrocardia include percutaneous clipping for severe mitral regurgitation using the MitraClip system (Abbott Vascular) and transcatheter aortic valve replacement for severe aortic valve stenosis.^7^, ^8^, ^9^ Additionally, the literature has a documented case of transapical, transcatheter atrioventricular valve-in-valve implantation in a patient with Fontan circulation with dextrocardia.^10^ These select cases underscore the limited interventions available overall for individuals with ACHD and dextrocardia. We believe that our case is the first to report the use of ViV-TTVR in a patient with CCTGA and situs inversus and is also the first instance of systemic atrioventricular valve-in-valve replacement in a patient with dextrocardia through a transseptal approach. Our report reinforces the excellent outcomes that can be achieved by transcatheter means in these patients.

## Conclusions

ViV-TTVR is a viable option for high–surgical risk patients with CCTGA and bioprosthetic systemic tricuspid valve degeneration.

## Funding Support and Author Disclosures

The authors have reported that they have no relationships relevant to the contents of this paper to disclose.

## Appendix

Video 1 Fluoroscopic Valve Positioning Precise positioning of the 29-mm Edwards Sapien 3 valve (Edwards Lifesciences) before deployment.

Video 2 Final Fluoroscopic View After Deployment of ViV-TTVR The final fluoroscopic view after deployment of the systemic tricuspid valve. Pressure measurements in the right-sided left atrium and systemic right ventricle revealed no residual pressure gradient across the systemic tricuspid valve.

Video 3 Final TEE View After Deployment of ViV-TTVR Final transesophageal echocardiographic (TEE) view demonstrated flow across the new valve with no perivalvular leak.
